# Adipokines as Prognostic Biomarkers in Multiple Myeloma: A Case–Control Study

**DOI:** 10.3390/medicina61112065

**Published:** 2025-11-20

**Authors:** Nóra Obajed Al-Ali, Dóra Csige, László Imre Pinczés, Katalin Farkas, István Rebenku, Andrea Domján, György Panyi, Zoltán Szekanecz, Gabriella Szűcs, Árpád Illés, László Váróczy

**Affiliations:** 1 Division of Hematology, Department of Internal Medicine, Faculty of Medicine, University of Debrecen, Nagyerdei krt. 98, 4032 Debrecen, Hungary; 2 Doctoral School of Clinical Medicine, University of Debrecen, Nagyerdei krt. 98, 4032 Debrecen, Hungary; 3 Division of Rheumatology, Department of Internal Medicine, Faculty of Medicine, University of Debrecen, 4032 Debrecen, Hungary; 4 Department of Biophysics and Cell Biology, Faculty of Medicine, University of Debrecen, 4032 Debrecen, Hungary

**Keywords:** multiple myeloma, prognostication, adiponectin, leptin, resistin, chemerin, adipsin, thrombospondin-1, paraoxonase-1, myeloperoxidase

## Abstract

*Background and Objectives:* Multiple myeloma (MM) remains an incurable plasma cell malignancy with heterogeneous clinical outcomes. Although current prognostic systems integrate biochemical and cytogenetic parameters, they do not fully capture disease complexity. Adipocytes within the bone marrow microenvironment secrete adipokines that regulate inflammation, metabolism, and immune interactions and may influence disease progression. This study aimed to assess circulating adipokines and related microenvironmental mediators as potential biomarkers of disease activity and treatment response in MM. *Materials and Methods:* In this case–control, cross-sectional study, the serum levels of eight adipokine-related molecules—adiponectin, leptin, resistin, chemerin, adipsin, thrombospondin-1 (TSP-1), paraoxonase-1 (PON-1), and myeloperoxidase (MPO)—were measured in 40 MM patients and 38 age- and sex-matched healthy controls. Enzyme-linked immunosorbent assays (ELISA) and bead-based multiplex immunoassays were used. Associations with prognostic markers (serum β_2_-microglobulin (sB2M), LDH, albumin, hemoglobin, renal function) and treatment response were analyzed using correlation and non-parametric statistical methods. *Results:* Compared to the controls, MM patients exhibited significantly higher circulating levels of adiponectin, resistin, chemerin, adipsin, TSP-1, and MPO, while leptin was decreased. Among clinical correlations, chemerin and PON-1 correlated positively with sB2M, TSP-1 correlated with LDH, and MPO correlated with M-protein and albumin. Resistin was lower in patients with renal impairment and an advanced disease stage. Adiponectin and TSP-1 were significantly lower in progressive disease compared to complete remission, suggesting their potential association with treatment response. *Conclusions:* This study demonstrates that multiple adipokines are dysregulated in MM and exhibit distinct associations with disease burden, renal function, and therapeutic response. Novel associations identified for TSP-1, PON-1, and adipsin highlight previously unrecognized microenvironmental pathways in MM biology. Adipokine profiling may complement established prognostic markers and provide new insights into the tumour microenvironment in MM.

## 1. Introduction

Multiple myeloma (MM) is a clonal plasma cell malignancy that accounts for approximately 10% of hematologic cancers and predominantly occurs in the elderly. Despite therapeutic advances, MM remains incurable in most cases, and prognosis varies widely among patients [1]. Conventional risk stratification relies on the International Staging System (ISS) and its revised version (R-ISS), incorporating serum β2-microglobulin (sB2M), lactate dehydrogenase (LDH), serum albumin levels, and cytogenetic abnormalities [2,3]. However, these markers do not fully account for disease heterogeneity, underscoring the need for novel biomarkers.

MM is identified as one of the malignancies positively correlated with obesity. The PROMISE trial demonstrated that obesity is associated with a 73% increase in the odds of developing monoclonal gammopathy of undetermined significance (MGUS) compared to individuals with normal weight, suggesting that obesity may facilitate the progression from MGUS to active MM [4]. Despite the clear epidemiological link between obesity and MM risk, the specific mechanisms by which obesity influences bone metabolism and disease progression in this plasma cell neoplasm remain inadequately explored [5,6].

The bone marrow microenvironment provides critical support for myeloma cell growth, migration, proliferation, survival, and drug resistance [1]. With advancing age, adipocytes become the predominant cellular component of the bone marrow. Bone marrow adipose tissue represents a distinct adipose depot that differs from white and brown adipose tissue in its origin, dietary responsiveness, phenotype, gene expression, and physiological functions [7]. It has gained particular attention for its role in MM pathogenesis due to firm interactions between marrow adipocytes and myeloma cells. Adipocytes are recognized as a metabolically active endocrine organ that produces and secretes a broad range of inflammatory mediators and adipokines, which are bioactive molecules that regulate inflammation, angiogenesis, metabolism, and immune responses [8,9]. Marrow adipocytes provide a protective niche for malignant cells by secreting various chemokines. Fluctuations in adipokine levels may exacerbate myeloma cell growth and survival while contributing to bone resorption [10]. Dysregulation of these adipokines has also been shown to facilitate carcinogenesis, including MM, by accelerating genomic instability, impairing DNA repair, and promoting immune escape and can directly provide a protective niche for malignant cells or impact disease progression in MM [11].

Given this background, adipokines represent promising candidates for biomarker discovery in MM. The aim of the current study was to identify the clinical value of these markers in a real-world population of MM patients. Therefore, we performed a case–control, cross-sectional study comparing the levels of eight adipokine-related markers (adiponectin, leptin, resistin, chemerin, adipsin, thrombospondin-1 [TSP-1], paraoxonase-1 [PON-1], and myeloperoxidase [MPO]) in MM patients and healthy controls and evaluated their associations with established prognostic parameters, disease stage, treatment response, and CRAB criteria (hypercalcaemia, renal failure, anemia, and bone lesions).

## 2. Materials and Methods

### 2.1. Study Population

For this case–control, cross-sectional study, we enrolled 40 patients affected by MM in a single centre (Division of Hematology, Department of Internal Medicine, University of Debrecen, Hungary) from November 2024 to December 2024 in whom serum samples were collected. Diagnosis of MM was based on the International Myeloma Working Group (IMWG) criteria [1]. Cases were documented using a case report form. Data on disease status, including age, sex, hemoglobin, LDH, renal function (creatinine, estimated glomerular filtration rate [GFR]), serum albumin, M-protein, serum-free light chains, ISS and R-ISS stage, fluorescence in situ hybridization (FISH) cytogenetics, treatment response categories, and CRAB features, were collected from medical records. FISH abnormalities associated with inferior prognosis encompassed t(4;14), t(14;16), and del(17p). Response assessment was performed according to IMWG guidelines, determining complete response (CR), very good partial response (VGPR), partial response (PR), no response (NR), and progressive disease (PD). Serum samples collected from 38 age- and sex-matched healthy controls without a history of hematologic malignancy were used for comparison with patient samples. All participants were included in accordance with the Declaration of Helsinki. Each participant received detailed information about the study and signed the patient information leaflet and the informed consent form prior to en-rollment. The study was approved by the Institutional Review Board of the University of Debrecen, Debrecen, Hungary.

### 2.2. Biomarker Measurements

We measured the serum levels of 8 analytes: adiponectin, leptin, resistin, chemerin, adipsin, TSP-1, PON-1, and MPO. Serum MPO, PON1, TSP-1, and chemerin levels were measured by the sandwich-type Enzyme-Linked Immunosorbent Assay (ELISA) method with the Human Myeloperoxidase ELISA Kit (AssayGenie, sensitivity: 0.089 ng/mL, Intra CV: 4.2%, Dublin, Ireland), Human PON1/Paraoxonase 1 ELISA Kit (AssayGenie, sensitivity: 9.375pg/mL, Intra-Assay: CV < 8%), Human Thrombospondin-1 ELISA Kit (AssayGenie, sensitivity: 1.875 ng/mL, Intra-Assay: CV < 8%), and Human Chemerin ELISA Kit (AssayGenie, sensitivity: 0.094 ng/mL, Intra-Assay: CV < 8%) in 1:100, 1:50, 1:115, and 1:40 dilutions, respectively. Serum adiponectin, adipsin, resistin, and leptin levels were evaluated by bead-based multiplex immunoassay followed by flow cytometry measurement using the LEGENDplex^TM^ Human Metabolic Panel 1 (4-plex) with a V-bottom plate (BioLegend^®^, San Diego, CA, USA, catalogue number: 740212) and the Novocyte 3000 RYB flow cytometer (ACEA Biosciences, Inc., Santa Clara, CA, USA), according to the manufacturer’s instructions. Intra-Assay Precision of LEGENDplex^TM^ Human Metabolic Panel 1 (4-plex): two samples with different concentrations of target proteins were analyzed in one assay with 16 replicates for each sample. The intra-assay precision is detailed in Supplementary Table S1. Analyte levels were correlated to other biomarkers as well as disease-specific features. Serum levels of LDH (normal: 135–220 U/L), sB2M (normal: 1.09–2.53 mg/L), free κ light chains (normal: 3.30–19.40 mg/L), free λ light chains (normal: 5.71–26.30 mg/L), albumin (normal: 60–72%), and creatinine were measured; GFR (normal: >90 mL/min/1.73 m^2^) was calculated; and hemoglobin (normal: 115–150 g/L for women and 130–165 g/L for men) levels were measured using LOINC Terminology Service (API) with HL7^®^ FHIR^®^ at the University of Debrecen. Monoclonal components were determined by immunofixation, plasma were deposited on a gel, and the proteins were sorted according to their size with an electrical current, followed by antigen deposition for each targeted type of immunoglobulin on the gel.

### 2.3. Statistical Analysis

Normality was assessed using the Kolmogorov–Smirnov test. Between-group differences (patients vs. controls; subgroups by ISS/R-ISS, CRAB, treatment response) were evaluated using independent *t*-tests or Mann–Whitney U tests as appropriate. Correlations between analytes and laboratory parameters (M-protein, sB2M, LDH, hemoglobin, albumin, free light chains, GFR) were analyzed using Pearson or Spearman correlation depending on the data distribution. Multigroup comparisons were conducted using ANOVA with Tukey’s post hoc test for normally distributed variables or Kruskal–Wallis with Dunn’s post hoc test otherwise. A *p*-value < 0.05 was considered statistically significant. Benjamini–Hochberg FDR 5% multiple-comparison corrections were performed. Analyses were performed using GraphPad Prism version 8.0.1 (GraphPad Software Inc., Boston, MA, USA).

## 3. Results

### 3.1. Patient Characteristics

The median age of the patient cohort was 69 years (range 39–81), and there were slightly more males than females. Most patients received an immunomodulatory drug (93%), a proteasome inhibitor (95%), and a steroid (100%), while six (15%) patients were treated with an anti-CD38 monoclonal antibody before sample collection. Most patients were in remission (CR or VGPR) at the time of data collection, while six (15%) of them experienced progressive disease (PD). The basic characteristics of the MM patients are summarized in Table 1.

### 3.2. Correlation with Patient Biology and Disease Characteristics

To assess the value of plasma adipokine levels as indicators of disease activity, we examined their associations with well-established patient- and disease-associated prognostic markers of MM. Age at sample collection was positively correlated with chemerin (r = 0.44, *p* = 0.005), TSP-1 (r = 0.39, *p* = 0.012) and PON-1 (r = 0.42, *p* = 0.008). These associations remained significant after Benjamini–Hochberg FDR 5% multiple-test correction, suggesting their substantial connection with age in the disease. No significant differences in analyte concentrations or other laboratory parameters were detected between male and female patients.

**Figure 1:**
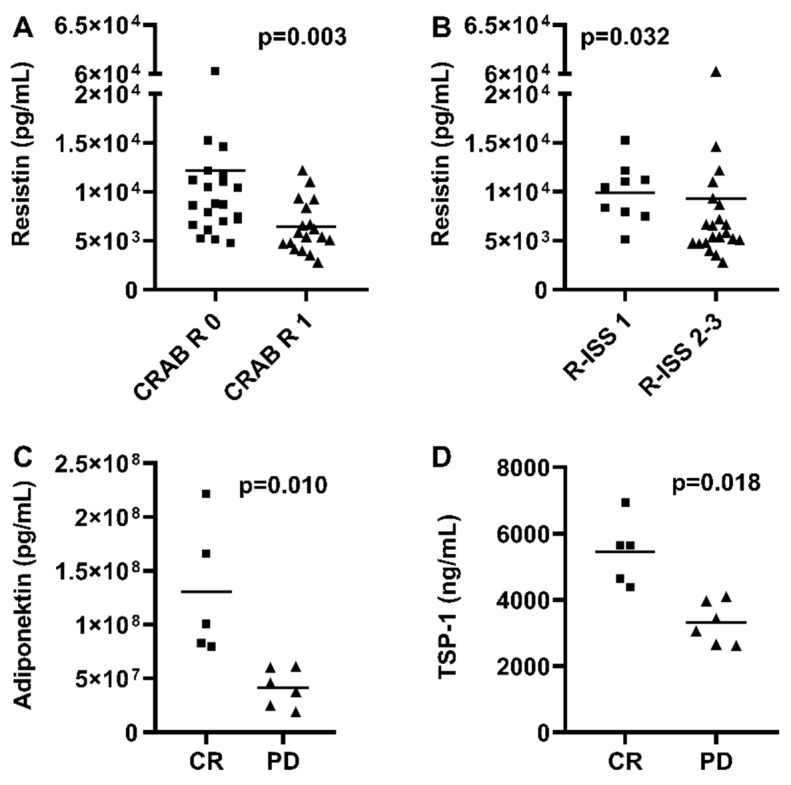
Serum adipokine levels in patients with renal failure according to CRAB criteria (**A**), R-ISS stage (**B**), and disease status (**C**,**D**). pg—picogram; mL—millilitre; ng—nanogram; TSP-1—thrombospondin-1; R-ISS—Revised International Staging System; CR—complete response; PD—progressive disease; CRAB—hypercalcaemia, renal failure, anemia, and bone lesions.

In patients with renal impairment according to CRAB criteria, resistin levels were significantly lower compared with those without renal involvement (8752 vs. 5624 pg/mL, *p* = 0.003) (Figure 1A). Resistin levels were increased in patients with R-ISS stage I disease compared to an advanced disease profile (R-ISS 2–3) (*p* = 0.048) (Figure 1B). When analyzing disease severity subgroups, resistin showed the following discriminatory capacity: within the CRAB classification, resistin distinguished patients with renal involvement from those without renal involvement, with an AUC of 0.78 (95% CI 0.63–0.92). Similarly, within the ISS staging system, resistin separated ISS stage 1 patients from ISS stage 2–3 patients, reaching an AUC of 0.71 (95% CI 0.54–0.89).

**Figure 2:**
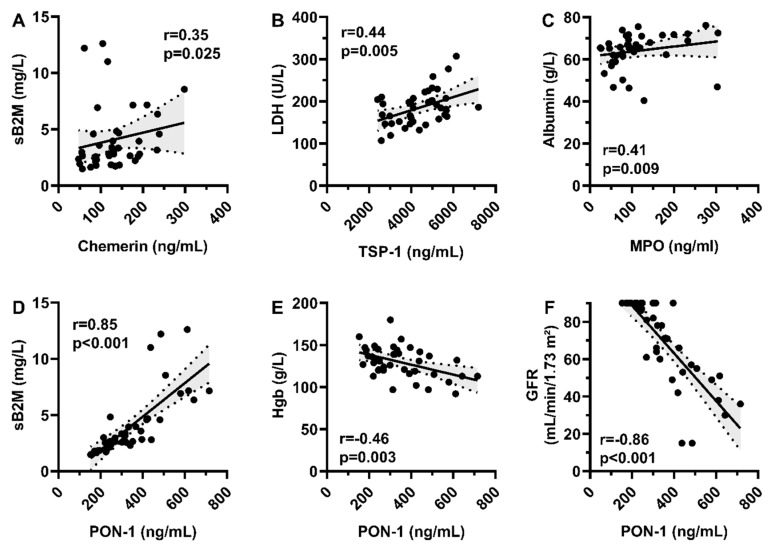
Correlations of adipokines and markers of disease activity (**A**–**F**). TSP-1—thrombospondin-1; MPO—myeloperoxidase; PON-1—paraoxanase; sB2M—serum beta-2-microglobulin; LDH—lactate dehydrogenase; Hgb—hemoglobin; GFR—glomerule filtration rate; mg—milligram; L—litre; U—unit; g—gram; mL—millilitre; min—minute; m—metre.

Chemerin levels correlated positively with sB2M (r = 0.35, *p* = 0.025) (Figure 2A), while TSP-1 correlated positively with LDH (r = 0.44, *p* = 0.005) (Figure 2B).

PON-1 demonstrated a positive correlation with sB2M (r = 0.85, *p* < 0.001), along with negative correlations with hemoglobin (r = −0.46, *p* = 0.003) and GFR (r = −0.86, *p* < 0.001) (Figure 2D–F).

MPO correlated positively with M-protein (r = 0.24, *p* = 0.043) and serum albumin levels (r = 0.41, *p* = 0.009) (Figure 2C).

Adiponectin, leptin, and adipsin did not significantly correlate with LDH, albumin, sB2M, hemoglobin, and renal impairment. No adipokine levels had significant associations with hypercalcemia, anemia, or the presence of bone lesions.

FISH-defined cytogenetic risk groups (standard vs. high risk) showed no significant differences in analyte levels, although sample size was limited due to data availability. Apart from resistin, no further differences in analyte levels were identified regarding ISS and R-ISS stages.

### 3.3. Correlation with Treatment Response

Out of 40 patients, 5 (12.5%) were in complete remission (CR) and 26 (65%) were in a very good partial remission (VGPR) at sample collection, while 3 patients experienced stable disease (SD) and 6 of them had progressive disease (PD). Response category analyses identified significantly lower levels of adiponectin and TSP-1 in patients with PD compared to patients in CR (4.15 vs. 13.03 × 10^7^, *p* = 0.010 and 3307 vs. 5455 ng/mL, *p* = 0.018, respectively) (Figure 1C,D).

### 3.4. Biomarker Levels Compared to Controls

**Figure 3:**
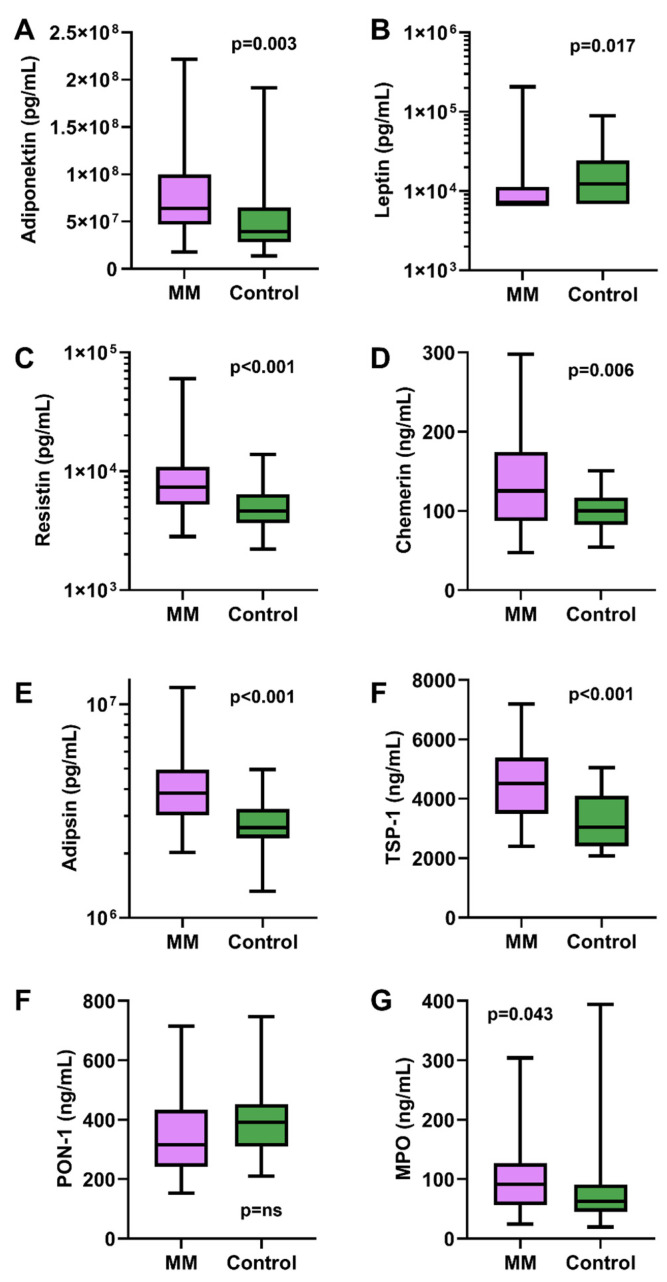
Serum adipokine levels of multiple myeloma patients and healthy controls (**A**–**G**). MM—multipe myeloma; TSP-1—thrombospondin-1; MPO—myeloperoxidase; PON-1—paraoxanase; mg—milligram; L—litre; U—unit; g—gram; mL—millilitre; min—minute; m—metre; ns—not significant.

Compared to the controls, MM patients had significantly higher mean levels for adiponectin (7.75 × 10^7^ vs. 5.21 × 10^7^ pg/mL, *p* = 0.003), resistin (9590 vs. 5471 pg/mL, *p* < 0.001), chemerin (131 vs. 101 pg/mL, *p* = 0.006), adipsin (4.25 × 10^6^ vs. 2.83 × 10^6^ pg/mL, *p* < 0.001), TSP-1 (4439 vs. 3261 ng/mL, *p* < 0.001), and MPO (110 vs. 95 ng/mL, *p* = 0.043) in the serum samples. In contrast, the mean leptin level was higher in healthy controls compared to the patients (2.69 × 10^4^ vs. 2.56 × 10^4^ pg/mL, *p* = 0.017). All seven differences remained significant after Benjamini–Hochberg FDR 5% multiple-test correction. Comparisons between patients and controls are depicted in Figure 3. The fold difference in the mean for adipokines in MM patients compared to the controls were modest (0.9–1.5), except from resistin, which had a 1.8-fold difference. PON-1 showed no significant difference between groups.

Based on the ROC analysis, adipsin (AUC = 0.78; 95% CI 0.67–0.88), TSP-1 (AUC = 0.78; 95% CI 0.67–0.88), and resistin (AUC = 0.76; 95% CI 0.65–0.86) demonstrated the strongest discriminatory ability between MM patients and healthy controls. Adiponectin (AUC = 0.70; 95% CI 0.58–0.81), leptin (AUC = 0.67; 95% CI 0.55–0.80), chemerin (AUC = 0.64; 95% CI 0.52–0.77), and MPO (AUC = 0.63; 95% CI 0.51–0.76) showed weaker yet statistically significant discrimination. Individual adipokine levels measured in patients and healthy controls are detailed in Supplementary Table S2.

## 4. Discussion

MM remains an incurable malignancy despite advances in therapy, and reliable prognostic markers are still limited. Current staging systems, such as ISS and R-ISS, incorporate laboratory parameters including sB2M, albumin, and LDH, yet they do not fully capture the biological heterogeneity of the disease. Increasing evidence suggests that the bone marrow microenvironment, particularly adipocytes and their secreted adipokines, plays a central role in supporting plasma cell survival, drug resistance, and disease progression. In this study, we comprehensively evaluated a panel of circulating adipokines and related microenvironmental mediators in patients with multiple myeloma and explored their associations with disease characteristics, treatment response, and clinical laboratory markers. Our findings provide further evidence that adipokine dysregulation is a relevant feature of MM pathobiology and highlight several candidate molecules that may contribute to disease progression or serve as potential biomarkers.

Adiponectin has emerged as a tumour-suppressive adipokine in MM, influencing plasma cell biology through metabolic and inflammatory signalling pathways [12]. Experimental data supports the anti-myeloma properties of adiponectin through the inhibition of AKT and NF-κB pathways [9]. Also, comparing patients with MGUS, smouldering MM, and overt MM, adiponectin levels were lower in smouldering and overt MM compared to MGUS [13]. Higher levels of pre-diagnostic adiponectin were associated with a lower risk of developing MM, particularly in the overweight population [14]. Altogether, adiponectin appears to act as a protective factor in the context of MM, with lower levels being associated with higher risk, more advanced disease, and more bone resorption. Contrary to previous epidemiological and preclinical data, we observed higher adiponectin concentrations in MM patients than in healthy controls. This discrepancy may reflect disease stage, treatment-related modulation of adipocyte function, or a compensatory systemic response to tumour-driven metabolic stress, attributable in whole or in part to the cross-sectional nature of our study. We are confident that treatment-related modulation is an important factor in elevated adiponectin levels. As documented in myeloproliferative disorders, patients with controlled disease had higher adiponectin levels than those with active disease who were not receiving treatment [15,16]. Similarly, in our patient population, adiponectin levels were significantly lower in progressive disease compared to complete remission, consistent with its proposed tumour-suppressive role.

Leptin is a pro-inflammatory peptide hormone regulating appetite and energy balance. Although most reports show elevated leptin in MM and link it to JAK/STAT and PI3K/AKT activation [17], we observed lower circulating levels in patients, while no correlations were identified between leptin and disease markers. This result is consistent with the previous finding that leptin concentrations decrease following treatment, which may be associated with an alteration in the metabolic state and the subsequent change in the chemokine profile [18]. These findings might also reflect treatment-related weight loss, altered adipose tissue mass, or catabolic states associated with advanced disease, illustrating the intricate interplay between systemic metabolism and MM biology. The complexity of leptin-mediated signalling is underlined by two separate prospective studies, in which no associations were identified between leptin levels and MM risk [12,19].

Resistin, an adipokine linked to insulin resistance and inflammation, has been associated with MM risk in prospective cohorts [20]. Beyond bone marrow adipocytes, it is expressed by osteoblasts and osteoclasts, implicating it in the regulation of bone turnover and remodelling [21]. Based on the largest meta-analysis, there is no difference in resistin levels between MM patients and healthy individuals [22]. The findings are contradictory, however, with smaller prospective studies suggesting that lower resistin levels may predispose to MM, particularly in men, potentially through dysregulated inflammatory signalling within the bone marrow microenvironment [20]. However, preclinical models have demonstrated that MM treatment results in significant decreases in Bcl-2 and Bcl-xL expression in myeloma cells, while the addition of resistin increases their expression [23]. This observation represents resistin-activated anti-apoptotic signalling pathways in myeloma cells, which may lead to an increase in resistin levels in a pre-treated patient population similar to ours. The paradoxical decrease in resistin among patients with renal impairment and advanced disease suggests that renal clearance and disease stage significantly modulate circulating levels. This complexity may explain inconsistencies in the literature and limits resistin’s utility as a standalone biomarker [22].

Chemerin plays a key role in microenvironmental signalling via the regulation of immune cell chemotaxis, adipocyte differentiation, and inflammation [24]. Data on chemerin in MM is limited, with one retrospective analysis of plasma samples from a randomized phase 3 clinical trial dataset [25]. Chemerin concentrations in patients were elevated compared to healthy controls, and serum levels rose in parallel with R-ISS stage. In our MM population, chemerin levels showed a similar, significant difference compared to healthy controls. Our confirmation of the correlation between chemerin and sB2M supports its role as a marker of tumour burden, and it is also consistent with the observation that both molecules are sensitive markers of decline in renal function [26,27]. Beyond a biomarker, chemerin may actively shape the tumour microenvironment by recruiting immune cells and modulating stromal signalling; processes that could influence plasma cell homing and growth.

Adipsin, also known as complement factor D (CFD), is a serine protease that plays a role in adipocyte biology, metabolic regulation, and the activation of the alternative complement pathway. It was demonstrated that adipocytes can activate autophagy and increase the expression of autophagy-related proteins like adipsin, thereby attenuating chemotherapy-induced caspase activation and apoptosis in myeloma cells [28]. A potential role of adipsin in the genesis of MM-associated bone disease has been postulated; however, it has not yet been studied as a disease marker. Although our study confirms elevated adipsin levels in MM, functional data remain sparse. Given its role in complement activation and chemotherapy resistance, further studies should investigate whether adipsin contributes directly to tumour survival or bone disease in vivo.

TSP-1 is a major regulator of latent TGF-β activation in the bone marrow microenvironment in MM. Through the modulation of TGF-β, TSP-1 has the potential to act as contributor to disease evolution and serve as a biomarker in MM. While no prospective studies have focused on TSP-1 levels in MM, high expression correlated with poor prognosis in several other cancer types [29].

In our patient population, TSP-1 was significantly elevated in MM patients compared to controls and correlated positively with LDH, a marker of progressive disease. Our finding that lower TSP-1 levels were associated with poor treatment response may reflect complex bidirectional roles in MM biology, where both excessive and deficient signalling can contribute to pathology. This observation aligns with the findings of Wu et al., who confirmed the downregulation of TSP-1 in relapsed or refractory MM compared to newly diagnosed patients [30].

PON-1 contributes to the elimination of free radicals, contributing to vulnerability to oxidative stress. Reduced PON-1 activity has been described in chronic inflammatory states, but its role in MM remains unexplored [31]. In a Turkish cohort, PON-1 levels were significantly lower in patients with multiple myeloma compared with the other groups, whereas no significant correlations were found between PON-1 levels and hemoglobin, creatinine, calcium, or albumin.

PON-1 showed a trend towards decreased plasma levels in MM patients compared to controls in our population, but the difference did not reach statistical significance. However, the strong correlations of PON-1 with sB2M, hemoglobin, and renal function indicate that oxidative stress and systemic inflammation are tightly linked to disease activity. This supports the notion that PON-1 could serve as a surrogate marker of metabolic stress in MM.

MPO, an oxidative enzyme expressed in myeloid cells, contributes to coping with oxidative stress, inflammation, and tissue injury and modulation of the immune microenvironment [32]. Enhanced MPO activity has been linked to the early stages of lung and breast carcinogenesis and has also been implicated in promoting tumour metastasis [33,34]. In hematological malignancies, MPO expression holds prognostic significance in B-cell acute lymphoblastic leukemia, where higher levels correlate with relapse and reduced event-free survival [35]. In MM, disease development is accompanied by increased production of MPO from myeloid cells, and elevated myeloid-derived MPO has been shown in vivo to contribute to MM progression [36]. In particular, increased MPO activity within the bone marrow microenvironment facilitates plasma cell homing and tumour expansion. Additionally, MPO enhances the expression of MM-supportive genes in bone marrow stromal cells and suppresses tumour-specific T-cell responses in vitro. Despite these observations, MPO has not yet been established as a biomarker in MM.

In our MM population, MPO levels were elevated compared to healthy controls and correlated positively with serum albumin and M-protein. Our findings, together with recent preclinical data, support a model in which MPO contributes to a tumour-permissive niche by promoting oxidative stress, suppressing anti-tumour immunity, and enhancing plasma cell migration. These findings contribute to the growing body of evidence that MPO is not just a bystander but an active contributor to MM growth.

Collectively, our findings highlight the potential of adipokine profiling as an adjunct to current risk stratification systems. Markers such as chemerin, TSP-1, PON-1, resistin, and MPO correlate with tumour burden and disease activity, while adiponectin and TSP-1 are linked to treatment response. These associations suggest that adipokine panels could complement conventional markers (sB2M, LDH, albumin) to refine prognostic models and inform personalized therapeutic strategies. Altogether, gathering real-world data in biomarker behaviour always conveys additional value. Regarding novelty, to the best of our knowledge, our trial is the first to describe that adiponectin and resistin levels may increase as a result of MM treatment and that adipsin is a potential biomarker of disease activity, while TSP-1 levels in peripheral blood are significantly elevated compared to healthy controls.

This study is limited by its modest sample size, cross-sectional study design, and lack of longitudinal follow-up. Differences in treatment status, body composition, and comorbidities may confound adipokine levels. Adipokine levels, which depend on treatment status, naturally influence the measured results, which can be considered a limitation of the study. However, the proportion of progressive patients corresponds to the response status of the patients currently followed for MM at our institution, thus representing real-world experience. Importantly, our study does not establish whether adipokine alterations are a cause or consequence of disease activity. Accordingly, some conclusions are based on biological plausibility and require further thorough functional work to confirm them. Also, mechanistic and interventional studies, including functional blockade or modulation of specific adipokines, will be necessary to determine their direct roles in MM biology.

Further investigation into the precise mechanisms by which metabolic changes, oxidative stress, and adipokine dysregulation contribute to disease biology in MM patients is essential to identify novel biomarkers and develop more effective interventions and targeted therapies for this challenging aspect of disease management.

## 5. Conclusions

This study demonstrates that multiple adipokines and microenvironment-derived mediators are dysregulated in MM and display distinct associations with disease activity and response to treatment. Notably, several factors not previously characterized in this context, including TSP-1, PON-1, and adipsin, emerged as potential indicators of disease biology and candidates for future research. Our results suggest that systemic metabolic and inflammatory signals reflect, and may actively shape, the tumour microenvironment and clinical course of MM. Further validation in larger, prospective cohorts will be essential to establish causality and evaluate the clinical utility of these biomarkers. Integrating adipokine profiling into clinical practice, together with genomic and immune parameters, could provide a more nuanced understanding of MM heterogeneity and open new avenues for biomarker-driven, microenvironment-targeted therapies.

## Figures and Tables

**Table 1:** **Patient** **characteristics.**

Variable		Data
Age, median, years (range)		69 (39–81)
Male/female, *n*		23/17
CRAB criteria, *n* (%)		
	Hypercalcaemia	6 (15)
	Renal failure	18 (45)
	Anemia	25 (63)
	Bone lesion	27 (68)
Cytogenetics, *n* (%)		
	Standard risk	26 (65)
	High risk	5 (13)
	n/a	9 (22)
R-ISS stage, *n* (%)		
	I	9 (22)
	II	11 (28)
	III	10 (25)
	n/a	10 (25)
Previous therapy, *n* (%)		
	Immunomodulatory drug	37 (93)
	Proteasome inhibitor	38 (95)
	Steroid	40 (100)
	Daratumumab	6 (15)
	Cyclophosphamide	10 (25)
	APSCT	30 (75)
Disease status, *n* (%)		
	CR	5 (13)
	VGPR/PR	26 (65)
	SD	3 (8)
	PD	6 (15)
Adipokines		
	Adiponectin, median, ×10^7^ ug/mL (range)	6.4 (1.8–22.2)
	Leptin, median, ng/mL (range)	6850 (6535–207,502)
	Resistin, median, ng/mL (range)	7336 (2819–60,272)
	Chemerin, median, ng/mL (range)	125 (48–298)
	Adipsin, median, ×10^6^ ug/mL (range)	3.8 (2.0–12.0)
	Thrombospondin-1, median, ug/mL (range)	4516 (2399–7189)
	Paraoxanase-1, median, ng/mL (range)	316 (153–715)
	Myeloperoxidase, median, mg/L (range)	91 (24–304)

R-ISS—Revised International Staging System; APSCT—autologous peripheral stem cell transplantation CR—complete response; VGPR—very good partial remission; PR—partial remission; SD—stable disease; PD—progressive disease; CRAB—hypercalcaemia, renal failure, anemia, and bone lesions.

## Data Availability

The datasets used and analyzed during the current study are available from the corresponding author on reasonable request.

## Supplementary Materials

The following supporting information can be downloaded at https://www.mdpi.com/article/10.3390/medicina61112065/s1. Table S1: Intra-Assay Precision of LEGENDplex TM Human Metabolic Panel 1; Table S2: Individual adipokine levels measured in patients (P) and healthy controls (C).
