# Supplementary material for: Adipokines as Prognostic Biomarkers in Multiple Myeloma: A Case–Control Study
Source: Medicina (Kaunas). 2025 Nov 20;61(11):2065. doi: 10.3390/medicina61112065 (PMC12654784; doi:10.3390/medicina61112065)
Supplement: Supplementary file 1 [file medicina-61-02065-s001.zip › medicina-3966530-supplementary.pdf]

Supplementary Materials to

Adipokines as Prognostic Biomarkers in Multiple Myeloma: A Case-Control Study

by

Nóra Obajed Al-Ali[1,2], Dóra Csige[2,3], László Imre Pinczés[1,2], Katalin Farkas[1,2], István Rebenku[4], Andrea Domján[3], György Panyi[4], Zoltán Szekanecz[3], Gabriella Szűcs[3], Árpád Illés[1,2], László Váróczy[1,2]

[1] Division of Hematology, Department of Internal Medicine, Faculty of Medicine, University of Debrecen, Debrecen, Hungary, [2] Doctoral School of Clinical Medicine, University of Debrecen, Debrecen, Hungary, [3] Division of Rheumatology, Department of Internal Medicine, Faculty of Medicine, University of Debrecen, Debrecen, Hungary, [4] Department of Biophysics and Cell Biology, Faculty of Medicine, University of Debrecen, Debrecen, Hungary

Corresponding author
Nóra Obajed Al-Ali
4032 Debrecen, Nagyerdei krt. 98., Hungary
obajed.nora@med.unideb.hu
phone: +36307731716
fax: +3652255112

Supplementary Table S1. Intra-Assay Precision of LEGENDplex TM Human Metabolic Panel 1

Two samples with different concentrations of target proteins were analyzed in one assay with 16 replicates for each sample.

pg – picogram, SD – standard deviation, %CV – coefficient of variability

Intra-Assay Precision				
**Analyte**	**Sample**	**Mean (pg/mL)**	**SD**	**%CV**
**Human Adiponectin**	**Sample 1**	**36.0**	**2.5**	**7%**
	**Sample 2**	**575.1**	**24.3**	**4%**
**Human Adipsin**	**Sample 1**	**41.2**	**2.4**	**6%**
	**Sample 2**	**678.9**	**44.8**	**7%**
**Human Leptin**	**Sample 1**	**32.3**	**2.1**	**6%**
	**Sample 2**	**613.1**	**32.3**	**5%**
**Human Resistin**	**Sample 1**	**36.1**	**2.4**	**7%**
	**Sample 2**	**664.4**	**48.3**	**7%**

Supplementary Table S2. Individual adipokine levels measured in patients (P) and healthy controls (C)

TSP-1 – thrombospondin-1, MPO – myeloperoxidase, PON-1 – paraoxanase, pg – picogram, mL – millilitre, ng – nanogram

ID	MPO (ng/ml)	PON-1 (ng/ml)	TSP-1 (ng/ml)	Chemerin (ng/ml)	Adiponectin (pg/ml)	Adipsin (pg/ml)	Leptin (pg/ml)	Resistin (pg/ml)
**P01**	50,89455	367,4905	3973,06	128,6632	37910753,5	4330533	12774,83	7013,379
**P02**	26,41533	391,2015	3701,137	96,4628	37412769,25	4128180	<6850	9339,61
**P03**	142,752	441,0305	5199,179	191,8544	56879860,5	5735776	<6850	15261,05
**P04**	60,42525	219,6775	4646,581	50,2934	100811934	2512584	9872,755	6544,603
**P05**	109,4613	269,2945	4092,166	56,5876	18057704,75	4058421	<6850	27421,85
**P06**	119,1188	352,4105	5405,811	169,135	73156837,5	5198196	8505,015	8799,197
**P07**	93,305	243,067	5645,333	87,259	83105896,75	2719317	9111,74	9228,965
**P08**	302,5648	617,496	7189,352	209,5874	138160173	4771451	14394,55	8703,948
**P09**	181,0103	315,762	5764,45	233,286	110185314,5	5521791	32381,22	14614,56
**P10**	77,802	579,677	6140,109	92,3908	25998348,5	4115569	<6850	8392,459
**P11**	24,453	171,2695	4081,132	88,892	103060330,3	3112226	<6850	8609,407
**P12**	304,2258	321,2855	3988,332	88,2794	59356047,5	5223345	27173,36	5394,543
**P13**	24,4865	268,919	4805,597	54,9936	137941047	3995549	<6850	60272,22
**P14**	199,228	195,745	3944,65	124,5904	66083043,5	3490622	<6850	6102,997
**P15**	232,4055	241,926	4809,869	181,3932	33098279,5	3776743	16676,64	5406,151
**P16**	91,634	341,2635	5309,613	90,0018	57331680,25	4450972	29308,86	3966,561
**P17**	53,47875	418,3415	5693,018	238,7272	50722846,75	7134804	29371,04	6211,131
**P18**	114,6528	436,944	5655,47	116,3652	166074934,3	12000000	6979,735	11018,65
**P19**	71,658	333,0555	3431,531	190,4856	45828241,25	4348814	<6850	5217,873
**P20**	172,0995	186,0435	3893,756	144,026	56934710,75	2651545	11671,78	4134,879
**P21**	232,1148	423,751	4092,735	142,3926	60210611,5	3160111	<6850	5052,066
**P22**	91,04635	396,11	5238,998	118,7392	78044320,5	2519963	<6850	7958,68
**P23**	34,13765	299,9045	3943,195	141,2386	134862351,5	3771910	<6850	7487,971
**P24**	44,97873	226,0405	5657,069	134,74	70109688,25	2744834	<6850	2818,517
**P25**	55,02975	221,6075	4683,122	85,695	86797837,75	3236395	<6850	10452,48
**P26**	46,47465	301,2975	4986,624	188,2598	54891321	3668782	8544,065	3525,823
**P27**	77,049	152,8955	2624,611	56,5198	61228560,5	2177696	<6850	4700,798
**P28**	55,692	611,3605	6942,263	104,5248	221607604,3	6234845	<6850	6652,807
**P29**	89,35375	481,6875	5028,789	82,5344	204543831,3	4263880	<6850	5134,647
**P30**	77,315	316,6055	2657,771	126,0286	18873933,25	3478655	<6850	12193,2
**P31**	104,434	249,053	5588,994	47,5918	103925412,5	3003220	8109,29	11918,77
**P32**	122,9375	485,704	2399,061	61,0978	83736685,5	10379712	207501,5	11195,73
**P33**	128,559	508,115	2753,169	297,8928	41424415,25	3896686	<6850	11024,8
**P34**	85,945	715,483	4989,051	175,8342	73281566,75	5660372	<6850	6632,631
**P35**	62,49775	312,749	3065,187	130,7692	24826306,25	2241429	9793,8	10471,64
**P36**	78,394	243,9145	2589,513	120,1288	97008008,75	3022590	<6850	4780,179
**P37**	108,1768	214,359	4386,399	130,2288	79671658,5	3109777	<6850	12182,75
**P38**	92,65225	246,259	3018,808	137,2078	60862575,75	3061365	32619,43	4756,149
**P39**	91,72025	180,149	2574,499	75,9468	33624563,25	2026460	<6850	7184,607

**P40**	275,2283	642,2215	2986,93	234,7076	50651284,75	4996786	9449,175	5841,53
**C01**	132,4793	313,593	4001,782	100,9178	191483000	2476124	7140	10043,1
**C02**	59,82075	323,2585	2853,863	136,2804	13761150	4900218	27776	4753,1
**C03**	97,16125	383,9175	2629,584	85,3778	75234450	2138847	<6850	4625,4
**C04**	55,2752	301,627	2349,387	108,3218	124895350	3012125	8080	3785,7
**C05**	72,84975	680,436	2115,253	150,65	35673950	2355789	<6850	4611,7
**C06**	203,3723	406,4565	2365,815	127,3966	19794850	1739356	<6850	7616,6
**C07**	86,57325	475,615	2914,848	91,157	76170050	2632435	23226	5328,3
**C08**	39,1817	399,7005	4306,785	109,32	37749600	4120370	53186	6344,9
**C09**	34,13643	339,127	2411,981	113,4624	57313600	2356659	<6850	3241,1
**C10**	67,86625	589,982	3118,662	137,3548	37210150	3763198	27921	7748,1
**C11**	61,64575	418,9995	3397,825	106,6206	63836400	4948119	78957	9208,4
**C12**	52,407	385,2925	2986,389	83,0268	68212650	2765074	24476	5707,5
**C13**	394,2898	512,921	2099,003	84,335	32254800	1792512	10052	2225,5
**C14**	32,568	369,9925	2201,721	100,328	137549400	3250391	13473	5870,2
**C15**	160,6648	444,193	2455,647	141,94	35337950	1441586	19518	7486,8
**C16**	22,3802	410,5735	2073,559	138,1638	75663200	2381963	23731	3650,8
**C17**	41,62693	375,6555	4358,65	121,3944	46637850	3564373	88667	13806,7
**C18**	323,0578	747,789	2369,707	144,6532	45506800	2401833	24967	6599,8
**C19**	51,02808	296,005	3263,965	58,5204	62671400	2420816	21057	3398,2
**C20**	65,52875	435,4675	2875,909	62,6256	96052900	3107683	18472	3465,1
**C21**	280,857	396,4375	2330,406	65,262	62025000	2374376	25941	3931,4
**C22**	82,1145	428,704	2143,1	90,7078	36149250	1329689	7520	5301,2
**C23**	85,7935	483,1125	2473,184	114,422	42166100	2809858	69149	12497,6
**C24**	63,02425	487,4015	3514,09	82,7462	26658350	2316133	21229	6121,3
**C25**	43,4342	416,466	2664,32	74,2946	72068700	2753019	11150	3738,4
**C26**	19,3633	385,636	3518,115	86,545	22897400	2287824	<6850	3117,5
**C27**	50,496	330,492	3378,712	78,1056	27350250	2408478	7250	5059,1
**C28**	53,73825	443,957	4222,133	82,393	52429200	4438453	17134	4650,7
**C29**	111,2155	601,716	3883,314	54,2086	26296950	2767963	21502	9710,1
**C30**	41,50233	317,754	4322,149	114,9772	14765600	2392465	6970	4330,3
**C31**	281,86	502,395	4271,767	115,2994	15419900	2266950	<6850	3675,2
**C32**	33,4477	210,5695	5051,789	144,1608	56602000	3232486	<6850	3899,5
**C33**	46,4304	237,6215	5032,337	88,818	37495550	3339748	7067	2505,1
**C34**	88,7605	254,4045	4791,383	59,1232	41210000	4365056	24318	5319,2
**C35**	45,75108	235,9675	2647,507	100,6144	22112900	2747573	<6850	2547,4
**C36**	59,915	255,652	3857,434	86,8522	28683700	2660737	<6850	3912,8
**C37**	87,34225	219,033	4060,633	74,944	28489100	2495548	<6850	4519,1
**C38**	64,02125	257,7005	4586,781	105,3422	35641050	2935461	<6850	3546,9
